# Slow reception and under-citedness in climate change research: A case study of Charles David Keeling, discoverer of the risk of global warming

**DOI:** 10.1007/s11192-017-2405-z

**Published:** 2017-05-16

**Authors:** Werner Marx, Robin Haunschild, Bernie French, Lutz Bornmann

**Affiliations:** 1 Max Planck Institute for Solid State Research, Heisenbergstraße 1, 70569 Stuttgart, Germany; 2 CAS Innovation LAB, CAS (Chemical Abstracts Service), a division of the American Chemical Society, 2540 Olentangy River Road, Columbus, OH 43202-1505 USA; 3 Division for Science and Innovation Studies, Administrative Headquarters of the Max Planck Society, Hofgartenstr. 8, 80539 Munich, Germany

**Keywords:** Bibliometrics, Climate change, Keeling curve, RPYS

## Abstract

The Keeling curve has become a chemical landmark, whereas the papers by Charles David Keeling about the underlying carbon dioxide measurements are not cited as often as can be expected against the backdrop of his final approval. In this bibliometric study, we analyze Keeling’s papers as a case study for under-citedness of climate change publications. Three possible reasons for the under-citedness of Keeling’s papers are discussed: (1) The discourse on global cooling at the starting time of Keeling’s measurement program, (2) the underestimation of what is often seen as “routine science”, and (3) the amount of implicit/informal citations at the expense of explicit/formal (reference-based) citations. Those reasons may have contributed more or less to the slow reception and the under-citedness of Keeling’s seminal works.

## Introduction

Charles David Keeling (1928–2005) started his scientific career as chemist at the Department of Geochemistry at Caltech, studying groundwater in the pristine Big Sur wilderness. Around 1956, Roger Revelle, director of the Scipps Institution of Oceanography near San Diego (California), invited Keeling to perform atmospheric carbon dioxide (CO_2_) measurements for the International Geophysical Year (IGY). Keeling had proposed that he could deploy a new analytical tool called infrared (IR) gas analyzer to perform continuous measurements of CO_2_ in air samples. The first analyzer was sent to Antarctica and a second one was prepared for installation aboard a research ship. In March 1958, an analyzer was installed on the top of Mauna Loa (Hawaii) with the intention to measure pristine air over the Pacific Ocean. Keeling soon detected strong seasonal variations in CO_2_ levels, oscillating from peak levels in the northern hemisphere spring (before the beginning of the growing season) and minimum levels in autumn. Since 1960, Keeling’s data revealed that CO_2_ levels were rising steadily in what became known as the “Keeling curve”. The data collection of Keeling’s measurement program is the longest continuous record of atmospheric CO_2_ in the world and shows that its concentration has grown from 315 ppm (parts per million) in 1958 to 408 ppm in May 2015 and is correlated to usage of fossil fuel as energy source.

Keeling is seen as the discoverer of the risk of global warming (Weart 1997). Keeling’s contribution to climate change research has been acknowledged and appreciated in numerous publications and press releases. A good example is a profound article presented under the rubric “National Historic Chemical Landmarks” of the American Chemical Society (ACS) entitled: “The Keeling curve: Carbon dioxide measurements at Mauna Loa”. The ACS states that “Keeling’s legacy includes a measurement program that endures to this day, providing an authoritative record of atmospheric CO_2_ concentrations that is a cornerstone of modern climate science”. We further get to know that “Keeling received numerous accolades during his career. He was elected a fellow of the American Academy of Arts and Sciences in 1986 and a member of the National Academy of Sciences in 1994. In 2002, Keeling was awarded the National Medal of Science, the nation’s highest award for lifetime achievements in science. In 2005, he received the Tyler Prize for Environmental Achievement for his data collection and interpretation” (ACS 2015). Further examples, which demonstrate the acknowledgement of Keeling’s lifework, are shown in the Appendix.

Keeling was a specific type of researcher, which can be seen as uncommon (e.g., he absolutely preferred an outdoor job). He was stimulated by Callendar (reading his papers) and by Plass (personal discussions). “He wants to measure CO_2_ in his belly … And he wants to measure it with the greatest precision and the greatest accuracy he possibly can” (Weart 2008, p. 34, cites Roger Revelle). This called for new and expensive instruments. Keeling ordered a gas analyzer from the only company, in which he “was able to get past a salesman and talk directly with an engineer” (Harris 2010, p. 7867). Keeling arranged many provisions to obtain representative data for pristine air.

One of his most important characteristics was his persistency and subject-specific determination: (1) He refused a job offer (for an indoor job in a dark cellar lab) from the Division of Meteorological Research in the Weather Bureau in Washington, DC. (2) Under Roger Revelle at Scripps Institution of Oceanography he refused to take only snapshots of the CO_2_ concentration every few years or every decade. (3) He continued to measure, although the up and down of the CO_2_ concentration measured within the first year seemed to confirm the previously measured inconsistent data—and the general impossibility to measure a baseline CO_2_ concentration. (4) He refused to replace his gas analyzer by newer instruments to avoid calibration problems and to ensure a highly consistent data row. In 1963, the work almost had to shut down. It was the so-called Sputnik-shock after the launch of the Soviet satellite in 1957 that boosted funding for all areas of science and education and allowed Keeling to continue his measurements at Mauna Loa.

In this study, we undertake a bibliometric analysis of Keeling’s publications. Bibliometrics is based on the assumption that important contributions to science receive high citation numbers. However, this is not always the case: Keeling’s contributions were definitely important for climate change research. However, we will demonstrate that his most important early publications are lower cited than expected in consideration of their importance for climate change. Their citation profiles are reminiscent of cases of slow reception and weak recognition. We will explore reasons for the under-citedness of Keeling’s papers.

## Methods

In our bibliometric analysis, we have investigated the lifework of Keeling from a quantitative (bibliometric) perspective. The bibliometric analysis has been performed in several steps by using different databases: 

1. In a first step, the profile of his publication history and the impact of his publications have been looked at. These analyses are based on Web of Science (WoS, Clarivate Analytics) data. We have searched for Keeling’s scientific publications and found 79 papers published between 1953 and 2004 (one paper appeared 2011, six years after his death). Is seems that Keeling always used his first and middle name initials—no relevant papers authored by C. Keeling could be found. Based on the publication set of 79 papers, we have established the WoS citation report.
2. In a second step, we broadened the perspective and analyzed whether slow reception (compared to the classical citation time pattern with a maximum of impact about three years after publication) and under-citedness are specific for Keeling’s papers or reflect general patterns of certain climate change papers. As in step 1, we used WoS data for this analysis.
3. In a third step, we focused on publications dealing with the measurement of the CO_2_ concentration in the earth’s atmosphere only and investigated Keeling’s citation impact on the field. The analysis is based on the search and retrieval functions of the databases offered by Chemical Abstracts Service (CAS), a division of the American Chemical Society (ACS). The CAS literature database (CAplus^SM^) covers scientific publications and patents since around the early 1800’s (including the references cited therein since the publication year 1996 of the database records). The CAS chemical substance database (CAS REGISTRY^SM^) contains all chemical species mentioned within disclosures in chemistry and related fields, identified and registered by the CAS Registry system. All compound records are associated with a unique CAS Registry Number^®^ (CAS RN^®^). Both databases are connected to each other via the CAS RN. The coverage and indexing of these subject specific databases are optimized in particular for searching literature dealing with chemical processes like the analysis of compounds.

In collaboration with CAS, we used both databases via the new platform of the Scientific and Technical Information Network (STN^®^) International. The CAplus publication records contain index terms (IT, keywords which are carefully selected and assigned by the database producer CAS). We searched for either one of the terms “anal?” or “occurr?” in combination (i.e. via the L operator of the STN retrieval language) with either of the terms “air” or “atmospher?” (? = truncation symbol to include “atmospheric” etc.) in combination with CO_2_ in the form of its CAS RN. This query returns all publications, which have words beginning with “anal” or “occurr” in their index terms, if the same index term group also contains “air” or a word starting with “atmospher”, and if the CAS RN of CO_2_ is listed in that index term group. The cited references within the resulting publication set (*n*_p_ = 2322 papers and *n*_r_ = 58,012 cited references) have been extracted.

The importance of individual papers within the literature of a specific research field can be detected by using a bibliometric method called “Reference Publication Year Spectroscopy” (RPYS, see Marx et al. 2014), preferably in combination with a recently developed tool named CRExplorer (http://www.crexplorer.net, see Thor et al. 2016). This method is based on the following findings: The analysis of the publication years of the references cited by all the papers in a specific research field shows that some publication years occur particularly frequently among the references. The years appear as pronounced peaks in the distribution of the reference publication years. The peaks are frequently based on single (or few) publications, which are highly cited compared to other publications published in the same year and are thus of specific significance to the research field in question (Marx et al. 2014). In recent years, several studies have been published, in which the RPYS was applied to examine the seminal papers of research fields (Marx et al. 2017a, b; Marx and Bornmann 2014; Comins and Hussey 2015a, b). The results were generally in accordance with the historical overviews of science historians.

The empirical part of the paper is followed by a theoretically oriented discussion of possible reasons for slow reception and weak recognition (i.e. under-citedness), in particular of Keeling’s papers.

## Results

### Publication and citation profile of Keeling

**Fig. 1:**
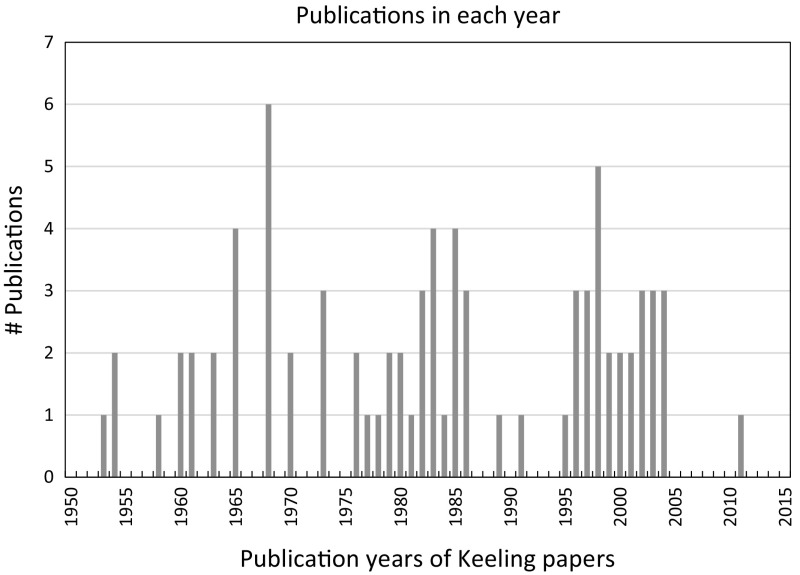
WoS diagram “published items in each year”: number of the Keeling papers as function of their publication years. *Source* WoS, date of searching: January 14, 2017

**Fig. 2:**
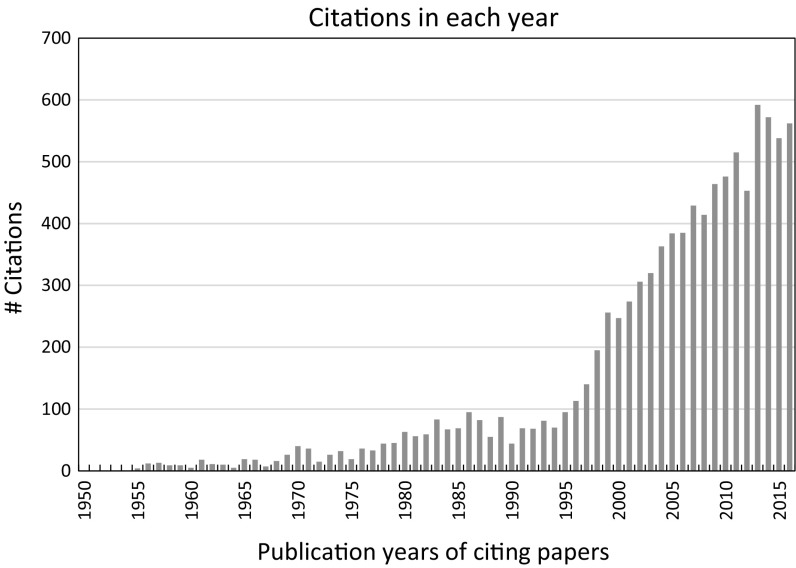
WoS diagram “citations in each year”: the overall number of citations as function of the years, in which the citing papers of Keeling’s publications were published. *Source* WoS, date of searching: January 14, 2017

We present in Fig. 1 the number of the Keeling papers as a function of their publication years. Figure 2 shows the overall number of citations as a function of the years, in which the citing papers of Keeling’s publications were published.

Keeling’s publication output is manageable. According to Fig. 1, he published on average 2–3 papers per year. According to Fig. 2, the overall citation impact of Keeling’s papers did not increase significantly before around 1995, when climate change research became a highly dynamic research field (see Haunschild et al. 2016).

**Fig. 3:**
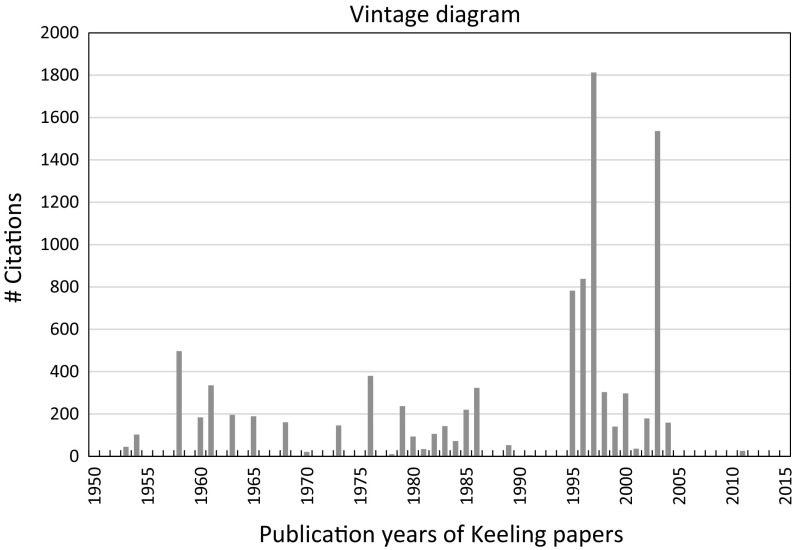
Number of citations as function of the publication years of the Keeling papers. *Source* WoS, date of searching: January 14, 2017

Since Fig. 2 does not show, which publications determined the massive impact since the mid-1990s, we investigated how the citation impact is distributed across the years of Keeling’s publications. Thus, Fig. 3 shows a vintage diagram with the number of citations as function of the publication years of his papers. This kind of diagram shows the citation impact contribution of publication years relative to each other (and thereby of the corresponding papers published in the specific years).

Although Keeling’s seminal papers (from 1960 and 1976) are his most important contributions to climate change research, they do not appear as the highest bars in the vintage diagram of Fig. 3. His 1995, 1996, 1997, and 2003 papers received significantly more citations. However, these papers (two of them with Keeling as co-author) do not deal primarily with measurements of the CO_2_ concentration in the atmosphere. They discuss the increased productivity of vegetation (based on satellite observations) and thereby the lengthening of the active growing season as a consequence of the recent global warming trend.

### Citation history of Keeling’s seminal papers

**Fig. 4:**
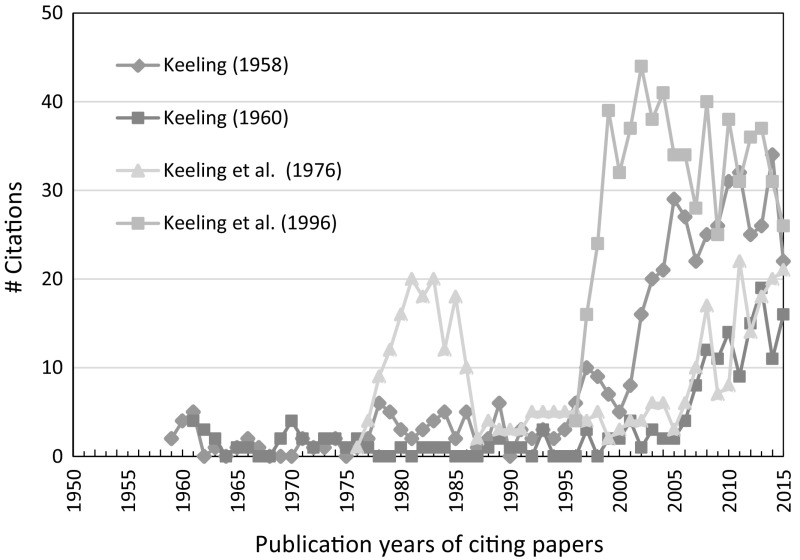
Time evolution of the citations per year of four Keeling papers. *Source* WoS

The WoS analyze function enables to establish the time evolution of the citations per year (the citation history) of a specific paper. We present in Fig. 4 the citation history of four selected Keeling papers, three early works including the seminal papers (from 1960 and 1976), and for comparison the highly cited 1996 paper.

Note that the 1958 paper deals with measurements in rural areas near the US Pacific coast. The 1958 paper and the 1976 paper, which presents for the first time the “Keeling curve”, attracted some attention between 1975 and 1985 and show a revival since around 2000, when current climate change research started to boom. The citation rate of the seminal 1960 paper was remarkably low before the 2000s. In strong contrast to the early works, the 1996 paper immediately attracted attention and its citation impact peaked a few years after publication. This corresponds to the citation time pattern of the majority of scientific papers.

In the case of papers that do not attract significant attention until decades after their publication, such as the Keeling (1960) paper, one refers to “slow reception” or “delayed recognition”. Also, these papers are called “sleeping beauties”. According to van Raan (2004), “a ‘Sleeping beauty in science’ is a publication that goes unnoticed (‘sleeps’) for a long time and then, almost suddenly, attracts a lot of attention (‘is awakened by a prince’)” (p. 461). Given the long time period of low citation rates and the unusually late impact, this definition formally fits the Keeling (1958, 1960) papers. However, delayed recognition not necessarily implies a high citation impact.

### Keeling’s papers are no isolated cases

**Fig. 5:**
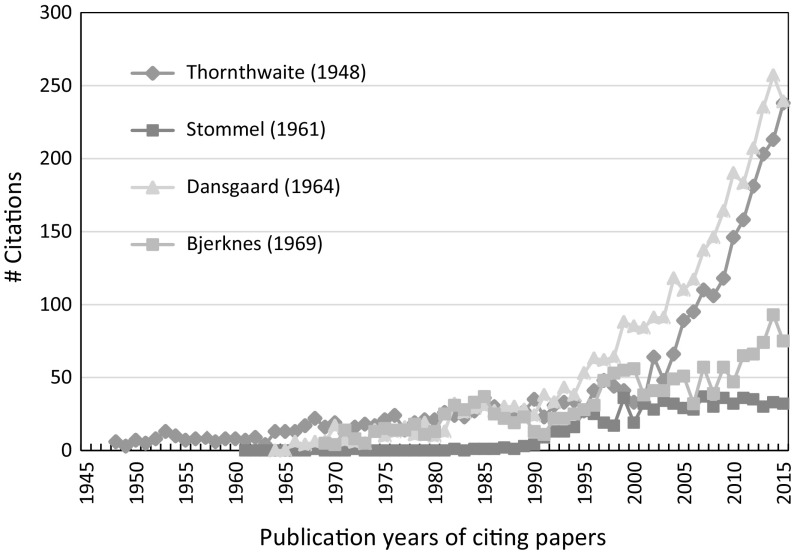
Citations per year of four highly cited early papers in the field of climate change research. *Source* WoS

The slow reception of Keeling’s papers is no exception in the climate change research literature. An inspection of the citation history of the papers assigned to the peaks in the RPYS spectrogram of the overall climate change research literature (see Marx et al. 2017b) showed that this phenomenon is not rare: Most of the papers analyzed are cases of delayed recognition; it seems to be a frequent phenomenon within climate change research. Good examples are the citation histories of Thornthwaite (1948), Stommel (1961), Dansgaard (1964), and Bjerknes (1969), which are presented in Fig. 5. Thornthwaite (1948) devised a moisture based climate classification system that is still in use worldwide. Stommel (1961) presented a model for the driving forces of the ocean circulation system. Dansgaard’s (1964) work is very important for the reconstruction of the past climate based on ice core samples. Bjerknes (1969) helped toward an understanding of El Niño Southern Oscillation (ENSO).

Haunschild et al. (2016) have shown that “the total number of papers dealing with climate change shows a strong increase: Within the time period 1991 to 2010, the number of climate change papers increased by a factor of ten and exhibits a doubling every 5–6 years” (p. 16). The exponential growth of climate change literature raises the number of potential citers and therewith the probability of relevant papers to be cited. A large community is an essential but not the only precondition for high citation counts. Other preconditions for example are importance, significance, and usefulness.

**Fig. 6:**
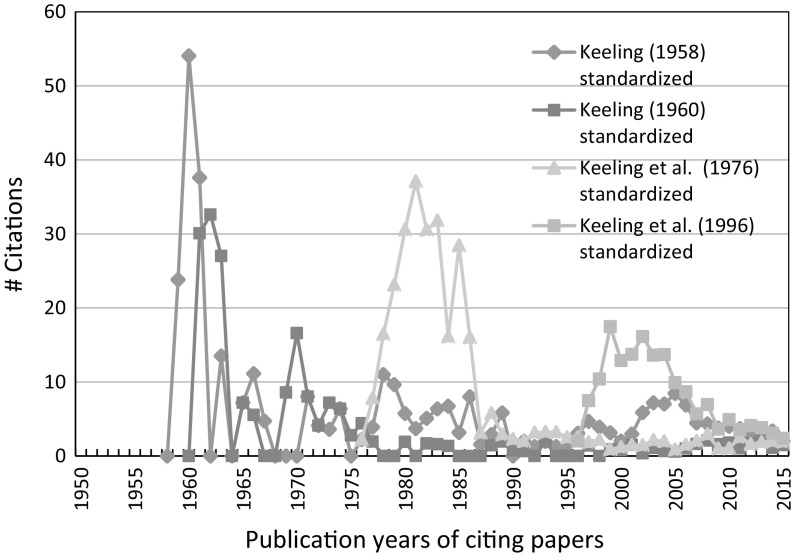
Standardized citations per year of four Keeling papers. *Source* WoS

Against the backdrop of the booming climate change literature, we standardized the citation numbers per year. We weighted the citation numbers with the number of publications per year dealing with climate change research. This reveals whether the Keeling papers have been perceived increasingly, despite the growing climate change research field. Figure 6 shows the “standardized” citation histories of the four papers presented in Fig. 4.

The standardization procedure was done as follows: (1) The annual number of climate change papers was determined using the WoS query ‘ti = (climat* OR paleoclimat* OR palaeoclimat* OR “global temperature*” OR “global warming” OR “greenhouse effect” OR “greenhouse gas*” OR “greenhouse warming”) and py = 1950–2015’. This query is based on a carefully constructed and tested query (Haunschild et al. 2016; Marx et al. 2017b). In contrast to the previous studies, here we searched only in the title field, because abstracts and keywords are not available reliably before 1991. (2) The number of citations from Fig. 4 is divided by the number of publications as determined in the previous step. (3) Finally, all standardized citations are multiplied with a factor of 1000.

According to Fig. 6, the four Keeling papers do not attract an increasing attention, when the booming climate change research is considered. Also, the overall citation impact, in particular of the 1960 paper, is not as high as one would expect in consideration of the basic importance of this work for climate change. After decades, such significant papers often have accumulated many thousands of citations. Hence, Keeling’s early works are cases of under-cited influential publications—not typical cases of sleeping beauties. Hu and Rousseau (2016) explain this phenomenon of under-citedness in an empirical study. They state that “a scientific contribution is not always what it looks as seen from a citation perspective” (p. 1081) and they present some cases of “fundamental work ahead of transformative research” (p. 1081). Inspecting the citing papers of Keeling (1960) reveals that 8 out of the 182 citing papers received more citations (ranging from 222 to 1427 citations at the date of searching: January 14, 2017) than the cited Keeling paper, although they have much shorter citation windows.

### The impact of Keeling on publications dealing with the measurement of the CO_2_ concentration in the earth’s atmosphere

**Fig. 7:**
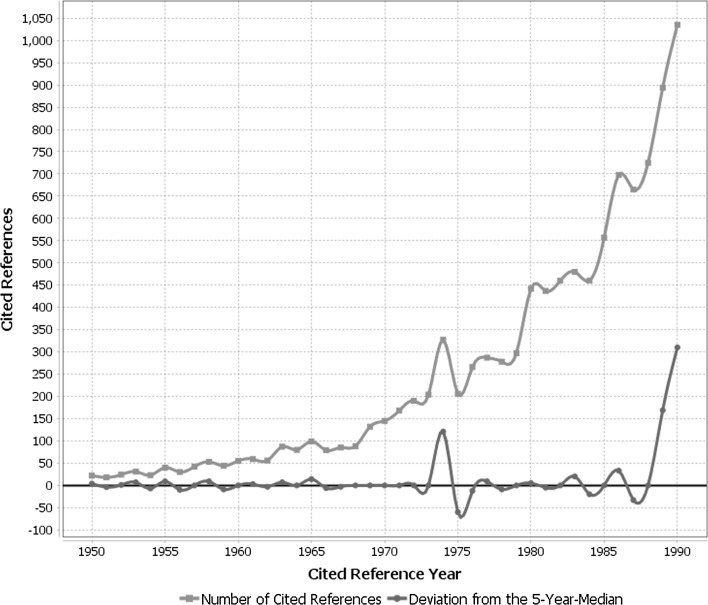
Annual distribution of cited references throughout the time period 1950–1990 (with 9013 cited references in this time period), which have been cited in papers dealing with the measurement of CO_2_ in the earth’s atmosphere. *Source* CAS, STN

The under-citedness of Keeling’s papers and their slow reception might be due to the fact that we have based the analyses on the entire WoS database, which is a multidisciplinary literature database. The specific contribution of his papers might become visible with a focus on the impact analysis of publications dealing with the measurement of the CO_2_ concentration in the earth’s atmosphere. For determining the contribution of Keeling’s landmark papers, we analyzed, which references have been most frequently cited by the papers dealing with the measurement of the CO_2_ concentration in the earth’s atmosphere. The RPYS spectrogram resulting from the analysis of the cited reference years via the CRExlorer software is shown in Fig. 7.

In agreement with the results reported above, the RPYS does not reveal the importance of Keeling’s works in the form of comparatively highly cited references within the subject specific literature: There are no pronounced peaks in the reference publication years 1960 and 1976, respectively, indicating the impact of Keeling’s early works on his Mauna Loa measurements of the atmospheric CO_2_ concentration. A second RPYS aproach based on a publication set searched in the multidisciplinary WoS via search terms (without the possibility to specify CO_2_ using its CAS RN) resulted in a quite similar spectrogram.

The only distinct peak in the spectrogram of Fig. 7 can be assigned to a paper by Weiss (1974). This publication delivers basic information concerning the solubility of CO_2_ in water and sea water, respectively. Studies about the solubility of CO_2_ in water concern the long discussed and most important question, which amount of the CO_2_ released by fossil fuel burning will remain in the atmosphere, and which portion will be solved in the oceans. In other words, such studies are connected to the question: Is the increasing CO_2_ concentration of any relevance with regard to an anthropogenic greenhouse effect—and by this to future climate?

The underestimation of precursor papers/forerunners has also been observed in other fields: Marx and Bornmann (2010, 2013) analyzed the evolution of the Big Bang theory and of the theory of Plate Tectonics. They discussed the comparatively low citation impact of some forerunners in the fields of astrophysics and geophysics, respectively. Hu and Rousseau (2016) mention three fundamental precursor papers in the fields of biochemistry and molecular biology that are most influential and highly cited but still under-cited in comparison with their relevance within the corresponding scientific field.

In consideration of the importance of Keeling’s and others’ papers for climate change research, we ask for the reasons of the slow reception (in terms of citation impact). Why did the scientific community underestimate the importance of a steadily rising CO_2_ level in the earth’s atmosphere for many decades? What are possible reasons for comparably low citation counts for Keeling’s landmark papers?

### Possible reasons for the under-citedness

#### The global cooling discussion

At the end of the nineteenth century and in the first half of the twentieth century, the scientists discussing the earth’s greenhouse effect and the role of atmospheric CO_2_ (Arrhenius, Callendar, Plass) were primarily interested in the past climate, particularly in the mystery of the ice ages (how could climate change at all?). Concerning the future climate, the dominant sentiment was that “warmer is better”. In the 1950s the popular press began to carry articles about global cooling, which was on the public agenda until the 1970s (Fleming 1998, pp. 131–133). And indeed, the average surface temperature of the northern hemisphere turned out to have significantly fallen between 1940 and 1980 (Jones et al. 1982).

Global cooling became an observable trend, stimulating speculations about the coming of a new ice age. Some experts began to ask whether the warming within the decades before 1940 had been an illusion. Later, scientists found out that the decline of the temperature was not seen in the southern hemisphere and concluded that the temperature drop after 1940 was largely due to a rise in industrial pollution (i.e. haze caused by aerosols), augmented by a long-term cycle in the Pacific Ocean. Further information can be found on the website of the science historian Spencer Weart (http://history.aip.org/climate/index.htm, in particular http://history.aip.org/climate/20ctrend.htm).

In consideration of falling average temperatures, it was hardly possible to take greenhouse warming seriously—in the public as well as in the scientific community. Keeling’s 1960 and 1976 papers appeared, when the scientific discourse about the risk of global warming had not yet emerged. We may assume that this is one reason for the comparatively low citation impact of Keeling’s early works within the first decades after their publication. This is the time span, in which scientific papers normally accumulate the largest portion of their citations. Later, Keeling’s early papers increasingly became historical papers, which basically have much less potential citers compared to classical research articles: Historical papers are predominantly cited by the much fewer authors of historical overviews and possibly in the introduction part of review articles.

#### Underestimation of “routine science”

Keeling’s scientific contributions are not typical for the scientific endeavor. They appeared as “routine” rather than novel science, thereby presumably limiting the probability of being cited. His colleague Euan Nisbet at the University of London mentioned in a biographical article in 2007: “Monitoring is science’s Cinderella, unloved and poorly paid” (Nisbet 2007, p. 789). And he added: “Many of Keeling’s problems came from the views of the scientific community itself. In situ work promises neither shiny rockets nor lucrative contracts. Monitoring does not win glittering prizes. Publication is difficult, infrequent and unread. Keeling’s extraordinary 1960 paper garnered citations slowly” (Nisbet 2007, p. 790).

Also, at the time when Keeling started his measurements, climate research was highly fragmented into a multitude of research disciplines and topics, and scientists acted independently from each other—thereby limiting the visibility of Keeling’s papers and the probability of citations. “The story of the discovery of global warming looks less like a processional march than like a scattering of groups wandering around an immense landscape. Many of the scientists involved are scarcely aware of one another’s existence” (Weart 2008, p. ix).

#### Implicit/informal citations

**Fig. 8:**
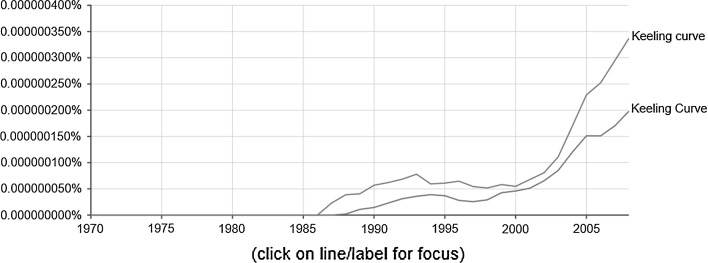
Annual mentions of “Keeling curve/Curve” in books searched under Google Ngram Viewer. The *y*-*axis* shows the yearly percentage of the 2-g “Keeling curve/Curve” of all the 2-g contained in the sample of books written in English and published within the time period 1871–2008. *Source* https://books.google.com/ngrams, date of searching: January 14, 2017

For many historical papers, a basic process limits the meaning of citation counts as a measure of scientific impact: “obliteration by incorporation”. This phenomenon was firstly described by the sociologist Robert K. Merton (1968). The process of obliteration affects seminal works offering novel ideas that are rapidly absorbed into the body of scientific knowledge. Such work is soon integrated into textbooks and becomes increasingly familiar within the scientific community. As a result of this absorption and canonization, the original sources fail to be cited in the reference lists. Seminal work is often cited by mentioning only the author’s name or name-based items (“implicit citations” or “informal citations”) instead of citing the source as a footnote (“formal citations”) (Marx and Cardona 2009; McCain 2012). The number of informal citations is often many times higher than the number of formal citations, in particular when the name of an author or his/her contribution has become a household word (like “Keeling curve”). Therefore, the works of Keeling are possibly subject to this phenomenon. According to Google Scholar, 1150 records mention the term “Keeling curve/Curve”. Google’s Ngram Viewer reveals a strong increase of book mentions since around 2000 (see Fig. 8).

The mentions of the Google Ngram Viewer are standardized with respect to the number of books per year, similar to our standardization method for establishing Fig. 6. The strong increase of the occurrences profile of the term “Keeling curve/Curve” under the Ngram Viewer compared to the WoS based citation profile of Keeling’s (1976) paper presented in Fig. 6 indicates that many formal citations are omitted. As a consequence, the overall impact of his pioneering paper cannot be entirely determined by merely counting their citations. Also, the book mentions of the term “Keeling curve/Curve” in Fig. 8 show the very late recognition: No mentions are found prior to 1987, more than ten years after the publication of Keeling’s (1976) paper, in which he presented the Keeling curve for the first time.

## Discussion

Our case study revealed the discrepancy between the current assessment of Keeling’s lifework and the comparatively low citation impact of his decisive papers on CO_2_ measurements in the earth’s atmosphere, which are undoubtedly most important for current climate change research and the future climate. We discussed three possible reasons for this discrepancy, which may have contributed more or less to the slow reception and the under-citedness of Keeling’s seminal works on the atmospheric CO_2_ concentration: (1) The discussion on global cooling at the starting time of Keeling’s measurement program, (2) the underestimation of what is often seen as “routine science”, and (3) the amount of implicit/informal citations at the expense of explicit/formal (reference-based) citations.

Keeling’s scientific contributions and the evolution of his citation impact show that, “sometimes, discovery comes slowly, not with a flash revelation but creepingly, as larger patterns emerge painfully from years of data” (Nisbet 2007, p. 789). Keeling’s measurement program also raises questions concerning past and present funding practices: “Although some scientists immediately recognized the importance of Keeling’s work, no agency felt responsible for funding a climate study that might run for many years. In 1963, the work almost had to shut down” (Weart 2008, p. 35). The Sputnik-shock after the launch of the Soviet satellite in 1957 boosted funding and allowed Keeling to continue his CO_2_ measurements at Mauna Loa. But “every few years, funding agencies sought to end his support because the work was judged to be ‘routine’ rather than novel research” (Harris 2010, p. 7870).

We learn from the study of Keeling’s contributions to climate change research that (1) a scientific contribution is not always what it looks as seen from a citation perspective, (2) bibliometric data should always be interpreted alongside expert knowledge, and (3) that funding agencies should reconsider their strategy and support long term projects.

## Appendix: Examples for demonstrating the acknowledgement of Keeling’s lifework

“Keeling’s work was far ahead of its time” (Nisbet 2007, p. 789).

“Keeling’s first report (Keeling 1960) is a landmark, documenting the seasonal cycle and, more gloomily, the annual rise of CO_2_” (Nisbet 2007, p. 789).

“Since then, the Keeling curve, the famous saw-toothed curve of rising CO_2_ concentrations, has become the environmental icon of the century” (Fleming 1998, p. 126).

“Keeling’s measurements … are the single most important environmental data taken in the twentieth century” (Harris 2010, p. 7865).

“Within a few years, Keeling’s inexorably rising CO_2_ curve was widely cited by scientific review panels and science journalists. It became the central icon of the greenhouse effect” (Weart 2008, p. 35).

“Keeling’s data put a capstone on the structure built by Tyndall, Arrhenius, Callendar, Plass, and Revelle and Suess. This was not quite the discovery of global warming. It was the discovery of the possibility of global warming” (Weart 2008, p. 37).
